# Supplementary material for: The language network responds robustly to sentences across tasks
Source: Imaging Neurosci (Camb). 2026 Jun 25;4:IMAG.a.1283. doi: 10.1162/IMAG.a.1283 (PMC13308801; doi:10.1162/IMAG.a.1283)
Supplement: Supplementary Material [file IMAG.a.1283_supp.pdf]

Supplementary Information

Behavioral Results

We found that accuracy decreased for tasks involving higher cognitive demands relative to the button press task. Interactions revealed substantial accuracy reductions relative to the button press task for the nonword condition paired with the hard memory probe ($\beta = -3.89$, SE = 0.34, $p < .001$), easy memory probe ($\beta = -0.92$, SE = 0.45, $p = .041$), sentiment ($\beta = -2.47$, SE = 0.57, $p < .001$), and comprehension question ($\beta = -2.60$, SE = 0.42, $p < .001$) tasks. For the sentence condition, accuracy significantly decreased when two sentences were presented compared to one ($\beta = -1.18$, SE = 0.31, $p < .001$). A significant interaction effect is found for the hard memory probe task and sentence condition ($\beta = 0.74$, SE = 0.26, $p = .005$). Additionally, sentence trials had overall higher accuracy compared to nonwords ($\beta = 0.67$, SE = 0.27, $p = .012$).

In line with accuracy findings, response times increased for all tasks compared to the button press task. Significant interactions were found between the nonword condition and the hard memory probe ($\beta = 0.43$, SE = 0.02, $p < .001$), easy memory probe ($\beta = 0.26$, SE = 0.02, $p < .001$), sentiment question ($\beta = 0.23$, SE = 0.11, $p = .043$), and comprehension question ($\beta = 1.30$, SE = 0.04, $p < .001$) tasks. For the sentence condition, response time significantly increased when two sentences were presented compared to one ($\beta = 0.08$, SE = 0.01, $p < .001$). A significant interaction effect is found for the hard memory probe task and sentence condition ($\beta = 0.03$, SE = 0.01, $p = .013$). Sentence trial response times were not significantly higher compared to nonword trials ($\beta = -0.02$, SE = 0.01, $p = .135$).

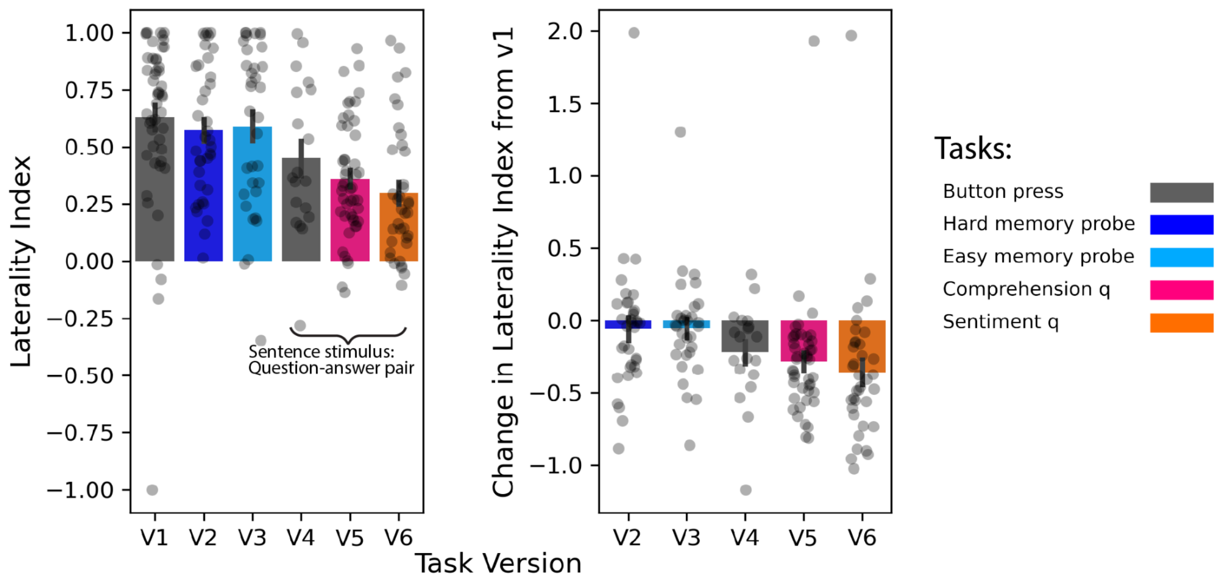

Supplemental Figure 1. *Laterality index across task versions and relative change from V1. Left: Mean laterality index for each task version. Each dot denotes a participant. Right: Change in laterality index for each task version relative to V1. Error bars denote the standard error.*

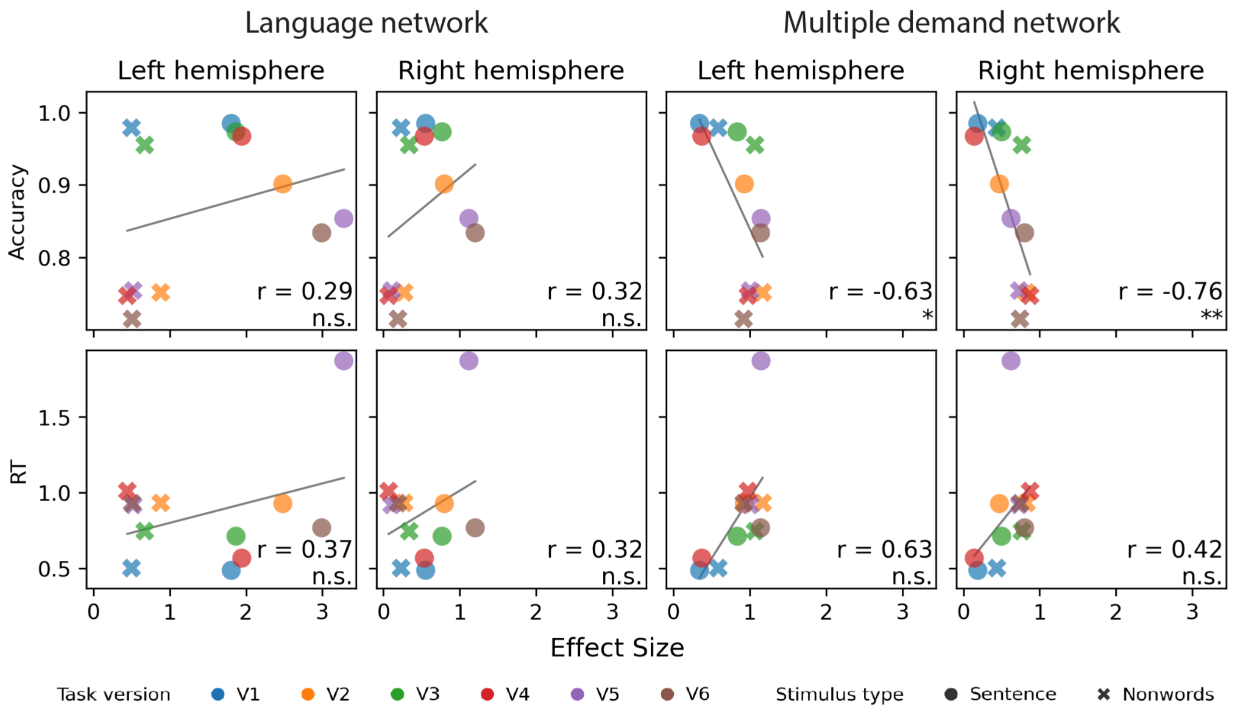

Supplemental Figure 2. *Average accuracy and response time versus the average effect sizes in the language and MD networks of language localizer tasks. FDR correction was conducted across the two hemispheres within each analysis and condition.*

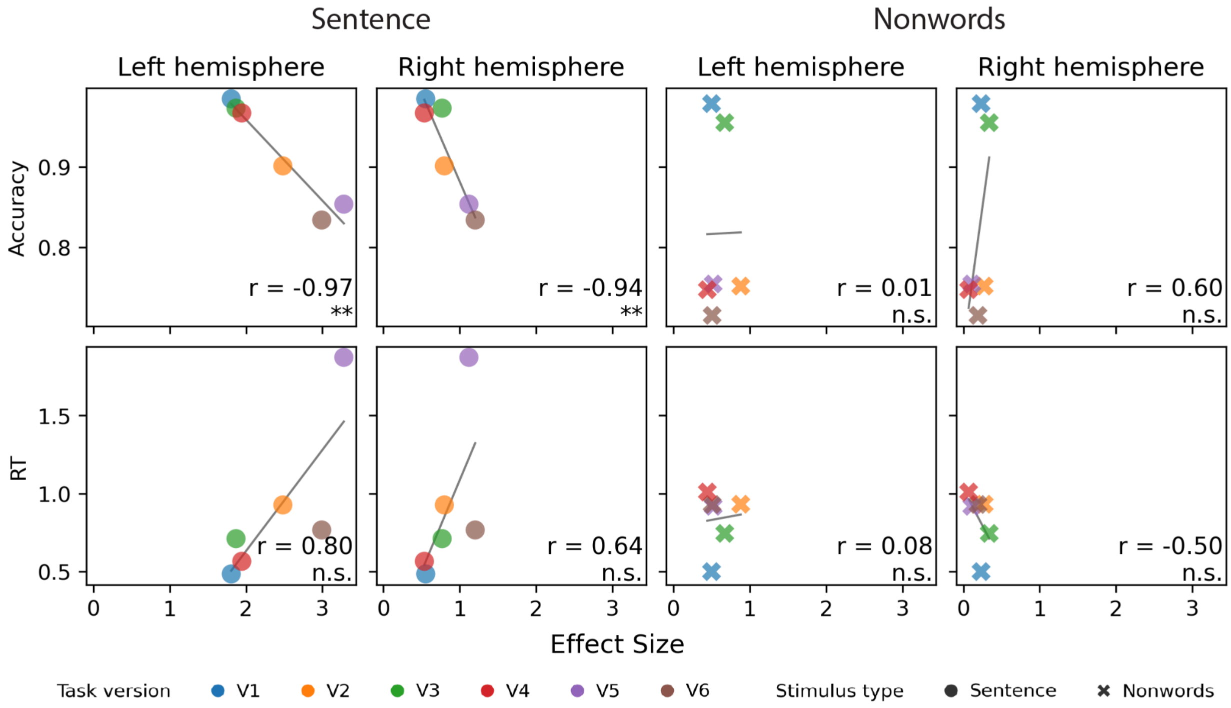

Supplemental Figure 3. *Average accuracy and response time versus the average effect sizes in the language networks of language localizer tasks by stimulus type. FDR correction was conducted across the two hemispheres within each analysis and condition.*

Supplemental Table 1. *Procedure and timing language localizer tasks. Each row denotes event sequences and durations of a trial.*

Version	Stimulus Type	Initial Blank	Stimulus Presentation	Probe	Post-event Blank	Total Trial Duration
V1 (button press)	Both	0.1 s	4.8 s (12 × 0.4 s)	0.4 s	0.7 s	6.0 s
V2 (hard memory probe)	Both	0.1 s	4.8 s	1.0 s	1.0 s	7.0 s
V3 (easy memory probe)	Both	0.1 s	4.8 s	1.0 s	1.0 s	7.0 s
V4 (button press)	Sentences	0.1 s	5.2 s (13 × 0.4 s)	0.4 s	0.6 s	9.0 s
V4 (hard memory probe)	Nonwords	0.1 s	4.8 s	2.6 s	0.4 s	8.0 s
V5 (comprehension q)	Sentences	0.1 s	4.8 s	2.6 s	0.4 s	8.0 s
V5 (hard memory probe)	Nonwords	0.1 s	4.8 s	2.6 s	0.4 s	8.0 s
V6 (sentiment q)	Sentences	0.1 s	5.2 s (13 × 0.4 s)	0.4 s	0.6 s	9.0 s
V6 (hard memory probe)	Nonwords	0.1 s	4.8 s	2.6 s	0.4 s	8.0 s

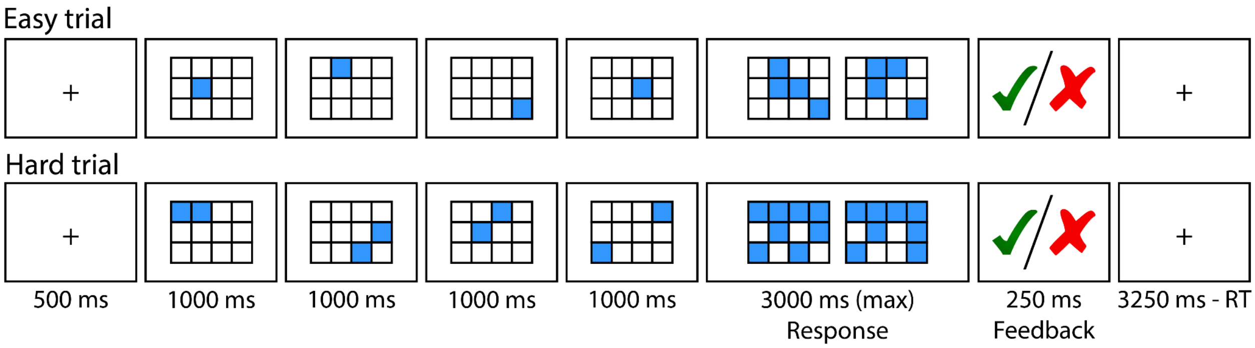

Supplemental Figure 4. *Procedure and timing for the spatial working memory task for localizing MD fROIs.*

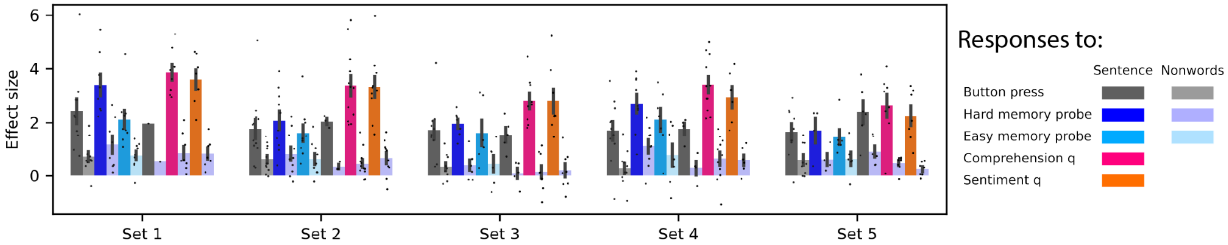

Supplemental Figure 5. *Responses of the core left-hemisphere language network to the sentence and nonword conditions by localizer material set.*

Supplemental Table 2. *Mixed-effect linear regression results for the left language networks on response magnitude by localizer task version. S - sentences, N - nonwords. Sentence (S) condition was used as the reference level (intercept) in the model.*

Localizer	V1		V2		V3		V4		V5		V6	
Regression Term	Beta	Sig	Beta	Sig	Beta	Sig	Beta	Sig	Beta	Sig	Beta	Sig
**(Intercept)**	**1.85**	***	**1.84**	***	**1.68**	***	**1.49**	***	**1.73**	***	**1.79**	***
Linguistic demand												
QuestionAns>OneSentence	**0.34**	*	**0.43**	*	0.17		**0.23**	*	**0.43**	**	**0.43**	**
Task demand												
**OneSentence:V2_MemoryAll>V1_ButtonPress**	**0.50**	***	**0.41**	**	**0.53**	***	**0.60**	**	**0.57**	***	**0.60**	***
OneSentence:V3_MemoryLast>V1_ButtonPress	0.01		0.00		-0.01		-0.32		0.06		0.03	
**QuestionAns:V5_ComprehensionQ>V4_ButtonPress**	**0.85**	***	**0.88**	**	**1.00**	***	**0.85**	**	**1.04**	***	**1.05**	***
**QuestionAns:V6_SentimentQ>V4_ButtonPress**	**1.14**	***	**1.14**	***	**1.44**	***	**1.21**	***	**1.38**	***	**1.42**	***
Stimulus type												
**OneSentence:V1_ButtonPress:N>S**	**-1.34**	***	**-1.44**	***	**-1.28**	***	**-1.12**	***	**-1.34**	***	**-1.28**	***
**OneSentence:V2_MemoryAll:N>S**	**-1.52**	***	**-1.64**	***	**-1.55**	***	**-1.72**	***	**-1.64**	***	**-1.75**	***
**OneSentence:V3_MemoryLast:N>S**	**-1.20**	***	**-1.28**	***	**-1.16**	***	**-0.90**	***	**-1.23**	***	**-1.12**	***
**QuestionAns:V4_ButtonPress:N>S**	**-1.61**	***	**-1.87**	***	**-1.49**	***	**-1.55**	***	**-1.70**	***	**-1.76**	***
**QuestionAns:V5_ComprehensionQ:N>S**	**-2.79**	***	**-3.04**	***	**-2.88**	***	**-2.73**	***	**-3.17**	***	**-3.22**	***
**QuestionAns:V6_SentimentQ:N>S**	**-2.52**	***	**-2.76**	***	**-2.50**	***	**-2.45**	***	**-2.84**	***	**-2.89**	***

Supplemental Table 3. *Mixed-effect linear regression results for the left language networks on response magnitude by fROI. S - sentences, N - nonwords. Sentence (S) condition was used as the reference level (intercept) in the model.*

ROI	IFGorb		IFG		MFG		AntTemp		PostTemp	
Regression Term	Beta	Sig.	Beta	Sig.	Beta	Sig.	Beta	Sig.	Beta	Sig.
**(Intercept)**	**1.64**	***	**1.87**	***	**2.54**	***	**1.17**	***	**1.73**	***
Linguistic demand										
QuestionAns>OneSentence	0.15		**0.38**	***	**0.61**	***	**0.18**	***	**0.38**	***
Task demand										
OneSentence:V2_MemoryAll>V1_ButtonPress	**0.35**	*	**0.87**	***	**0.81**	***	0.08		**0.30**	**
OneSentence:V3_MemoryLast>V1_ButtonPress	-0.18		0.21		0.07		-0.03		-0.07	
**QuestionAns:V5_ComprehensionQ>V4_ButtonPress**	**1.52**	***	**1.89**	***	**1.55**	***	**0.62**	***	**0.84**	***
**QuestionAns:V6_SentimentQ>V4_ButtonPress**	**1.14**	***	**1.49**	***	**1.05**	***	**0.55**	***	**0.68**	***
Stimulus type										
**OneSentence:V1_ButtonPress:N>S**	**-1.22**	***	**-1.44**	***	**-1.74**	***	**-1.03**	***	**-1.27**	***
**OneSentence:V2_MemoryAll:N>S**	**-1.47**	***	**-1.98**	***	**-1.91**	***	**-1.17**	***	**-1.50**	***
**OneSentence:V3_MemoryLast:N>S**	**-1.16**	***	**-1.40**	***	**-1.43**	***	**-0.99**	***	**-1.21**	***
**QuestionAns:V4_ButtonPress:N>S**	**-1.57**	***	**-1.65**	***	**-1.97**	***	**-1.47**	***	**-1.80**	***
**QuestionAns:V5_ComprehensionQ:N>S**	**-3.20**	***	**-3.69**	***	**-3.53**	***	**-2.03**	***	**-2.60**	***
**QuestionAns:V6_SentimentQ:N>S**	**-2.79**	***	**-3.33**	***	**-3.10**	***	**-1.96**	***	**-2.48**	***

Supplemental Table 4. *Mixed-effect linear regression results for the left and right language networks on response magnitude. S - sentences, N - nonwords. Sentence (S) condition was used as the reference level (intercept) in the model.*

Hemisphere	Left hemisphere				Right hemisphere			
Regression Term	Beta	SE	p value		Beta	SE	p value	
**(Intercept)**	**1.79**	**0.27**	**<.001**	***	**0.57**	**0.11**	**<.001**	***
Linguistic demand								
QuestionAns>OneSentence	**0.34**	**0.06**	**<.001**	***	-0.01	0.04	0.740	
Task demand								
OneSentence:V2_MemoryAll>V1_ButtonPress	**0.48**	**0.09**	**<.001**	***	0.06	0.07	0.387	
OneSentence:V3_MemoryLast>V1_ButtonPress	0.01	0.11	0.959		0.13	0.10	0.189	
**QuestionAns:V5_ComprehensionQ>V4_ButtonPress**	**1.28**	**0.13**	**<.001**	***	**0.59**	**0.10**	**<.001**	***
**QuestionAns:V6_SentimentQ>V4_ButtonPress**	**0.97**	**0.16**	**<.001**	***	**0.72**	**0.12**	**<.001**	***
Stimulus type								
**OneSentence:V1_ButtonPress:N>S**	**-1.34**	**0.09**	**<.001**	***	**-0.37**	**0.06**	**<.001**	***
**OneSentence:V2_MemoryAll:N>S**	**-1.61**	**0.09**	**<.001**	***	**-0.50**	**0.06**	**<.001**	***
**OneSentence:V3_MemoryLast:N>S**	**-1.24**	**0.09**	**<.001**	***	**-0.48**	**0.06**	**<.001**	***
**QuestionAns:V4_ButtonPress:N>S**	**-1.69**	**0.14**	**<.001**	***	**-0.52**	**0.10**	**<.001**	***
**QuestionAns:V5_ComprehensionQ:N>S**	**-3.00**	**0.12**	**<.001**	***	**-1.14**	**0.09**	**<.001**	***
**QuestionAns:V6_SentimentQ:N>S**	**-2.72**	**0.16**	**<.001**	***	**-1.21**	**0.13**	**<.001**	***

Supplemental Table 5. *Mixed-effect linear regression results for the left and right MD networks on response magnitude. S - sentences, N - nonwords. Sentence (S) condition was used as the reference level (intercept) in the model.*

Hemisphere	Left hemisphere				Right hemisphere			
Regression Term	Beta	SE	p value		Beta	SE	p value	
(Intercept)	**0.37**	**0.15**	**0.025**	*	0.20	0.12	0.113	
Linguistic demand								
QuestionAns>OneSentence	0.05	0.07	0.421		**-0.19**	**0.06**	**0.002**	**
Task demand								
**OneSentence:V2_MemoryAll>V1_ButtonPress**	**0.51**	**0.09**	**<.001**	***	**0.28**	**0.11**	**0.013**	*
**OneSentence:V3_MemoryLast>V1_ButtonPress**	**0.45**	**0.10**	**<.001**	***	**0.38**	**0.11**	**0.002**	**
**QuestionAns:V5_ComprehensionQ>V4_ButtonPress**	**0.72**	**0.10**	**<.001**	***	**0.59**	**0.12**	**<.001**	***
**QuestionAns:V6_SentimentQ>V4_ButtonPress**	**0.77**	**0.13**	**<.001**	***	**0.85**	**0.16**	**<.001**	***
Stimulus type								
**OneSentence:V1_ButtonPress:N>S**	**0.23**	**0.07**	**0.001**	**	**0.24**	**0.07**	**0.001**	**
**OneSentence:V2_MemoryAll:N>S**	**0.22**	**0.08**	**0.005**	**	**0.31**	**0.08**	**<.001**	***
**OneSentence:V3_MemoryLast:N>S**	**0.18**	**0.08**	**0.021**	*	**0.19**	**0.08**	**0.021**	*
**QuestionAns:V4_ButtonPress:N>S**	**0.73**	**0.13**	**<.001**	***	**0.90**	**0.13**	**<.001**	***
QuestionAns:V5_ComprehensionQ:N>S	-0.13	0.08	0.121		0.11	0.09	0.186	
QuestionAns:V6_SentimentQ:N>S	-0.16	0.10	0.122		-0.02	0.12	0.853	

Supplemental Table 6. *Mixed-effect linear regression results for the left and right DMN networks on response magnitude. S - sentences, N - nonwords. Sentence (S) condition was used as the reference level (intercept) in the model.*

Hemisphere	Left hemisphere				Right hemisphere			
Regression Term	Beta	SE	p value		Beta	SE	p value	
(Intercept)	0.08	0.13	0.530		-0.13	0.09	0.175	
Linguistic demand								
QuestionAns>OneSentence	-0.01	0.08	0.852		-0.01	0.07	0.906	
Task demand								
**OneSentence:V2_MemoryAll>V1_ButtonPress**	**-0.33**	**0.08**	**<.001**	***	**-0.36**	**0.08**	**<.001**	***
OneSentence:V3_MemoryLast>V1_ButtonPress	-0.07	0.09	0.419		0.02	0.08	0.845	
**QuestionAns:V5_ComprehensionQ>V4_ButtonPress**	**-0.30**	**0.10**	**0.003**	**	**-0.39**	**0.09**	**<.001**	***
QuestionAns:V6_SentimentQ>V4_ButtonPress	-0.25	0.14	0.076		**-0.35**	**0.13**	**0.008**	**
Stimulus type								
OneSentence:V1_ButtonPress:N>S	0.05	0.07	0.466		**0.15**	**0.06**	**0.017**	*
OneSentence:V2_MemoryAll:N>S	-0.02	0.08	0.802		0.13	0.07	0.072	
OneSentence:V3_MemoryLast:N>S	-0.06	0.08	0.436		0.01	0.07	0.859	
**QuestionAns:V4_ButtonPress:N>S**	**-0.51**	**0.13**	**<.001**	***	**-0.32**	**0.12**	**0.006**	**
QuestionAns:V5_ComprehensionQ:N>S	-0.09	0.08	0.280		0.13	0.07	0.057	
QuestionAns:V6_SentimentQ:N>S	-0.11	0.11	0.306		0.15	0.08	0.080	

Supplemental Table 7. *Mixed-effect linear regression results for the left and right language networks on spatial correlation. S - sentences, N - nonwords.*

Hemisphere	Left hemisphere				Right hemisphere			
Regression Term	Beta	SE	p value		Beta	SE	p value	
**(Intercept)**	**0.53**	**0.11**	**0.002**	**	**0.31**	**0.07**	**0.003**	**
Stimulus type								
N.S>N.N	-0.01	0.01	0.302		-0.01	0.01	0.531	
**S.S>N.N**	**0.36**	**0.01**	**<.001**	***	**0.15**	**0.01**	**<.001**	***
Linguistic demand								
**N.S:OneSentence.QuestionAns>OneSentence.OneSentence**	**-0.08**	**0.00**	**<.001**	***	**-0.02**	**0.00**	**<.001**	***
S.S:OneSentence.QuestionAns>OneSentence.OneSentence	**-0.02**	**0.00**	**<.001**	***	0.01	0.00	0.280	
**S.S:QuestionAns.QuestionAns>OneSentence.OneSentence**	**0.19**	**0.01**	**<.001**	***	**0.14**	**0.01**	**<.001**	***
Task demand								
N.N:ButtonPress.Task>ButtonPress.ButtonPress	0.02	0.01	0.175		0.01	0.01	0.445	
**N.N:Task.Task>ButtonPress.ButtonPress**	**0.18**	**0.01**	**<.001**	***	**0.16**	**0.01**	**<.001**	***
N.S:ButtonPress.Task>ButtonPress.ButtonPress	0.01	0.01	0.188		0.00	0.01	0.846	
**N.S:Task.Task>ButtonPress.ButtonPress**	**0.07**	**0.01**	**<.001**	***	**0.08**	**0.01**	**<.001**	***
S.S:ButtonPress.Task>ButtonPress.ButtonPress	-0.01	0.01	0.218		**0.03**	**0.01**	**<.001**	***
**S.S:Task.Task>ButtonPress.ButtonPress**	**0.14**	**0.01**	**<.001**	***	**0.16**	**0.01**	**<.001**	***

Supplemental Table 8. *Mixed-effect linear regression results for the left and right MD networks on spatial correlation. S - sentences, N - nonwords.*

Hemisphere	Left hemisphere				Right hemisphere			
Regression Term	Beta	SE	p value		Beta	SE	p value	
**(Intercept)**	**0.40**	**0.07**	**<.001**	***	**0.34**	**0.05**	**<.001**	***
Stimulus type								
N.S>N.N	-0.01	0.03	0.757		-0.02	0.03	0.470	
S.S>N.N	**0.11**	**0.03**	**<.001**	***	0.02	0.03	0.576	
Linguistic demand								
**N.S:OneSentence.QuestionAns>OneSentence.OneSentence**	**0.02**	**0.01**	**<.001**	***	**0.03**	**0.01**	**<.001**	***
S.S:OneSentence.QuestionAns>OneSentence.OneSentence	**0.02**	**0.01**	**0.041**	*	-0.01	0.01	0.314	
**S.S:QuestionAns.QuestionAns>OneSentence.OneSentence**	**0.18**	**0.01**	**<.001**	***	**0.11**	**0.01**	**<.001**	***
Task demand								
**N.N:ButtonPress.Task>ButtonPress.ButtonPress**	**0.08**	**0.02**	**0.001**	**	**0.05**	**0.03**	**0.035**	*
**N.N:Task.Task>ButtonPress.ButtonPress**	**0.34**	**0.02**	**<.001**	***	**0.29**	**0.03**	**<.001**	***
N.S:ButtonPress.Task>ButtonPress.ButtonPress	**0.05**	**0.01**	**0.001**	***	0.01	0.01	0.338	
**N.S:Task.Task>ButtonPress.ButtonPress**	**0.26**	**0.01**	**<.001**	***	**0.22**	**0.01**	**<.001**	***
S.S:ButtonPress.Task>ButtonPress.ButtonPress	0.02	0.01	0.173		**0.03**	**0.02**	**0.025**	*
**S.S:Task.Task>ButtonPress.ButtonPress**	**0.25**	**0.02**	**<.001**	***	**0.26**	**0.02**	**<.001**	***

Supplemental Table 9. *Mixed-effect linear regression results for the left and right DMN networks on spatial correlation. S - sentences, N - nonwords.*

Hemisphere	Left hemisphere				Right hemisphere			
Regression Term	Beta	SE	p value		Beta	SE	p value	
**(Intercept)**	**0.22**	**0.05**	**<.001**	***	**0.23**	**0.04**	**<.001**	***
Stimulus type								
N.S>N.N	-0.04	0.04	0.365		-0.06	0.04	0.107	
**S.S>N.N**	**0.14**	**0.04**	**0.001**	***	**0.10**	**0.04**	**0.015**	*
Linguistic demand								
N.S:OneSentence.QuestionAns>OneSentence.OneSentence	0.00	0.01	0.670		0.01	0.01	0.246	
S.S:OneSentence.QuestionAns>OneSentence.OneSentence	**0.04**	**0.01**	**0.004**	**	-0.01	0.01	0.709	
**S.S:QuestionAns.QuestionAns>OneSentence.OneSentence**	**0.18**	**0.02**	**<.001**	***	**0.12**	**0.02**	**<.001**	***
Task demand								
N.N:ButtonPress.Task>ButtonPress.ButtonPress	-0.03	0.04	0.330		-0.03	0.04	0.336	
**N.N:Task.Task>ButtonPress.ButtonPress**	**0.13**	**0.04**	**<.001**	***	**0.14**	**0.04**	**<.001**	***
N.S:ButtonPress.Task>ButtonPress.ButtonPress	-0.02	0.02	0.334		-0.03	0.04	0.336	
**N.S:Task.Task>ButtonPress.ButtonPress**	**0.07**	**0.02**	**<.001**	***	**0.14**	**0.04**	**<.001**	***
S.S:ButtonPress.Task>ButtonPress.ButtonPress	0.02	0.02	0.283		-0.01	0.01	0.709	
**S.S:Task.Task>ButtonPress.ButtonPress**	**0.15**	**0.02**	**<.001**	***	**0.12**	**0.02**	**<.001**	***

Supplemental Table 10. *Results of post-hoc linear mixed-effects regression tests on fROI overlap in the left and right language networks.*

Hemisphere	Left hemisphere				Right hemisphere			
Contrast	Beta	SE	p value		Beta	SE	p value	
Within-participant.Same-task vs Within-participant.Different-task								
V1	**0.06**	**0.01**	**<.001**	***	-0.01	0.01	1.000	
**V2**	**0.06**	**0.01**	**<.001**	***	**0.03**	**0.01**	**<.001**	***
V3	0.02	0.01	0.117		-0.01	0.01	1.000	
**V4**	**0.07**	**0.01**	**<.001**	***	**0.09**	**0.01**	**<.001**	***
**V5**	**0.16**	**0.01**	**<.001**	***	**0.18**	**0.01**	**<.001**	***
**V6**	**0.13**	**0.01**	**<.001**	***	**0.16**	**0.01**	**<.001**	***
Within-participant.Different-task vs Between-participant.Same-task								
**V1**	**0.33**	**0.01**	**<.001**	***	**0.19**	**0.01**	**<.001**	***
**V2**	**0.39**	**0.01**	**<.001**	***	**0.22**	**0.01**	**<.001**	***
**V3**	**0.36**	**0.01**	**<.001**	***	**0.21**	**0.01**	**<.001**	***
**V4**	**0.38**	**0.01**	**<.001**	***	**0.22**	**0.01**	**<.001**	***
**V5**	**0.40**	**0.01**	**<.001**	***	**0.23**	**0.01**	**<.001**	***
**V6**	**0.39**	**0.01**	**<.001**	***	**0.24**	**0.01**	**<.001**	***

Supplemental Table 11. *Results of post-hoc linear mixed-effects regression tests on fROI overlap by ROI in the left language network.*

ROI	IFGorb		IFG		MFG		AntTemp		PostTemp	
Contrast	Beta	Sig.	Beta	Sig.	Beta	Sig.	Beta	Sig.	Beta	Sig.
Within-participant.Same-task vs Within-participant.Different-task										
**V1**	**0.07**	***	**0.07**	***	**0.07**	***	**0.02**	*	**0.04**	***
**V2**	**0.07**	***	**0.08**	***	**0.08**	***	**0.04**	***	**0.05**	***
V3	-0.03		**0.05**	*	0.03		0.01		**0.03**	*
**V4**	**0.10**	***	**0.06**	**	**0.08**	***	**0.06**	***	**0.06**	***
**V5**	**0.19**	***	**0.15**	***	**0.18**	***	**0.14**	***	**0.14**	***
**V6**	**0.12**	***	**0.13**	***	**0.16**	***	**0.11**	***	**0.13**	***
Within-participant.Different-task vs Between-participant.Same-task										
**V1**	**0.27**	***	**0.33**	***	**0.34**	***	**0.32**	***	**0.41**	***
**V2**	**0.30**	***	**0.40**	***	**0.40**	***	**0.36**	***	**0.47**	***
**V3**	**0.28**	***	**0.38**	***	**0.36**	***	**0.35**	***	**0.43**	***
**V4**	**0.30**	***	**0.38**	***	**0.40**	***	**0.34**	***	**0.46**	***
**V5**	**0.33**	***	**0.41**	***	**0.41**	***	**0.38**	***	**0.46**	***
**V6**	**0.31**	***	**0.41**	***	**0.41**	***	**0.37**	***	**0.46**	***

Supplemental Table 12. Multivariate pattern analysis decoding accuracies. SVM: Support Vector Machine, kNN: k-Nearest Neighbors, LR: Logistic Regression. Bonferroni correction for multiple comparisons within each network and classification task.

Network	Testing Condition	Correlation		SVM		kNN		LR	
		Accuracy	Sig.	Accuracy	Sig.	Accuracy	Sig.	Accuracy	Sig.
Within-condition training: stimulus type classification									
Language (LH)	Button press	0.67	***	0.73	***	0.66	***	0.69	***
	hard memory probe	0.70	***	0.77	***	0.68	***	0.72	***
	Easy memory probe	0.69	***	0.73	***	0.65	***	0.68	***
Language (RH)	Button press	0.61	***	0.64	***	0.60	***	0.63	***
	hard memory probe	0.66	***	0.68	***	0.64	***	0.66	***
	Easy memory probe	0.59	***	0.62	***	0.59	***	0.62	***
MD	Button press	0.55	**	0.57	***	0.56	***	0.59	***
	hard memory probe	0.58	***	0.60	***	0.57	***	0.65	***
	Easy memory probe	0.56	***	0.56	***	0.54	**	0.60	***
Within-condition training: task classification									
Language (LH)	Sentence	0.46	***	0.51	***	0.41	***	0.48	***
	Nonwords	0.39	***	0.48	***	0.38	**	0.42	***
Language (RH)	Sentence	0.43	***	0.49	***	0.42	***	0.45	***
	Nonwords	0.37	**	0.45	***	0.39	**	0.42	***
MD	Sentence	0.44	***	0.52	***	0.44	***	0.56	***
	Nonwords	0.42	***	0.52	***	0.40	***	0.53	***
Across-condition training: stimulus type classification									
Language (LH)	Button press	0.66	***	0.70	***	0.65	***	0.67	***
	hard memory probe	0.70	***	0.75	***	0.65	***	0.69	***
	Easy memory probe	0.69	***	0.70	***	0.65	***	0.68	***
Language (RH)	Button press	0.62	***	0.65	***	0.63	***	0.64	***
	hard memory probe	0.66	***	0.67	***	0.61	***	0.63	***
	Easy memory probe	0.61	***	0.62	***	0.59	***	0.58	***
MD	Button press	0.56	**	0.58	***	0.56	***	0.60	***
	hard memory probe	0.58	***	0.59	***	0.56	**	0.63	***
	Easy memory probe	0.56	**	0.56	***	0.56	***	0.58	***
Across-condition training: task classification									
Language (LH)	Sentence	0.38	**	0.44	***	0.36	*	0.38	***
	Nonwords	0.38	**	0.43	***	0.37	**	0.40	***
Language (RH)	Sentence	0.39	***	0.45	***	0.38	**	0.41	***
	Nonwords	0.39	***	0.45	***	0.39	***	0.41	***
MD	Sentence	0.41	***	0.51	***	0.39	***	0.53	***
	Nonwords	0.42	***	0.49	***	0.41	***	0.54	***

Supplemental Table 13. Binomial mixed-effects model predicting decoding accuracy. Fixed effects included training type, system (LH language vs. MD), testing group, and their interactions. Random intercepts were included for participant and classifier type.

Regression Term	Estimate	SE	*z*	p value	95% CI
Stimulus type decoding					
(Intercept)	-0.06	0.11	-0.59	0.558	[-0.27, 0.15]
Language_LH:AcrossCondition:TestOnN	-0.39	0.05	-8.60	<.001	[-0.48, -0.30]
Language_LH:WithinCondition:TestOnN	-0.26	0.05	-5.77	<.001	[-0.35, -0.17]
Language_LH:AcrossCondition:TestOnS	-0.39	0.05	-8.58	<.001	[-0.48, -0.30]
Language_LH:WithinCondition:TestOnS	-0.09	0.05	-2.10	0.036	[-0.18, -0.01]
MD:AcrossCondition:TestOnN	-0.10	0.05	-2.15	0.032	[-0.19, -0.01]
MD:WithinCondition:TestOnN	-0.05	0.05	-1.17	0.240	[-0.14, 0.04]
MD:AcrossCondition:TestOnS	-0.10	0.05	-2.30	0.021	[-0.19, -0.02]
Task decoding					
(Intercept)	0.27	0.08	3.43	<.001	[0.11, 0.42]
Language_LH:AcrossCondition:TestOnV1	0.46	0.06	8.06	<.001	[0.35, 0.57]
Language_LH:WithinCondition:TestOnV1	0.53	0.06	9.25	<.001	[0.42, 0.64]
Language_LH:AcrossCondition:TestOnV2	0.58	0.06	10.10	<.001	[0.47, 0.69]
Language_LH:WithinCondition:TestOnV2	0.68	0.06	11.62	<.001	[0.56, 0.79]
Language_LH:AcrossCondition:TestOnV3	0.50	0.06	8.82	<.001	[0.39, 0.62]
Language_LH:WithinCondition:TestOnV3	0.56	0.06	9.66	<.001	[0.44, 0.67]
MD:AcrossCondition:TestOnV1	0.03	0.06	0.55	0.582	[-0.08, 0.14]
MD:WithinCondition:TestOnV1	0.01	0.06	0.13	0.896	[-0.10, 0.12]
MD:AcrossCondition:TestOnV2	0.10	0.06	1.86	0.062	[-0.01, 0.21]
MD:WithinCondition:TestOnV2	0.14	0.06	2.54	0.011	[0.03, 0.25]
MD:AcrossCondition:TestOnV3	0.01	0.06	0.14	0.890	[-0.10, 0.12]
